# Correlates of Loneliness in Older Korean Americans: Interactive Effects of Negative Family Interactions

**DOI:** 10.1093/geroni/igaa057.2170

**Published:** 2020-12-16

**Authors:** Nan Sook Park, Yuri Jang, David Chiriboga, Soondool Chung

**Affiliations:** 1 University of South Florida, Tampa, Florida, United States; 2 University of Southern California, Los Angeles, California, United States; 3 Ewha Womans University, Seoul, Republic of Korea

## Abstract

This study examined factors affecting the feelings of loneliness among older Korean Americans. Data were drawn from a survey with older Korean Americans aged 60 or over (N = 2,150) in five states (California, New York, Texas, Hawaii, and Florida), conducted during 2017−2018. In hierarchical multiple regression models, loneliness was regressed on five blocks of variables: (1) demographic/health (age, gender, education, financial status, chronic conditions, and physical disabilities); (2) immigration-related (length of stay in the U.S., and acculturation); (3) social engagement (having meals alone, family network, friend network, activity participation, and community engagement); (4) negative family interactions; and (5) interactions of negative family interactions with social engagement variables. A significant interaction was found in the relationship between friend network and negative family interactions: the impact of negative family interactions on loneliness was buffered by friend network. Implications of findings were discussed regarding working with older immigrants with limited social networks.

